# Development of intraoperative fluoroscopic protocols to avoid rotational malalignment during nailing of femoral fractures: a step-by-step guideline using the lesser trochanter profile or true lateral view

**DOI:** 10.1007/s00590-026-04810-1

**Published:** 2026-06-24

**Authors:** Daniel J. Vermue, Pim J. Bongers, Kaj Ten Duis, Job N. Doornberg, Ruurd L. Jaarsma, Mostafa El Moumni, Nils Jan Bleeker, Raul G. Plomp, Frank F. A. IJpma, 3D Surgery Consortium., J. de Haas, T. Otto, M. Hogervorst, E. Fennema, M. El Moumni, R. G. Plomp, H. de Haan, J. Hoekstra, M. Keasberry, W. Spierenburg, R. Koster, S. Zwerver

**Affiliations:** 1 Department of Trauma Surgery, University Medical Center Groningen, Groningen, The Netherlands; 2 Orthopaedic Trauma Surgery, Flinders Medical Centre, Bedford Park, Australia; 3 Department of Orthopaedic Surgery, Amsterdam University Medical Centers, Amsterdam, The Netherlands

**Keywords:** Femur, Fractures, Malrotation, Fluoroscopy, Lesser trochanter, True lateral technique

## Abstract

**Purpose:**

Rotational malalignment (≥ 15°) occurs in up to 35% of femoral fractures after intramedullary nailing, and standardized fluoroscopy protocols to prevent malrotation are lacking. We developed and evaluated two easy-to-use intraoperative fluoroscopy techniques to reduce malrotation.

**Methods:**

A human specimen study was performed to compare two standardized fluoroscopy protocols—the lesser trochanter profile and the true lateral view—with current clinical practice. A mid-shaft femoral fracture was created in a full-body cadaver, and an unlocked intramedullary nail was inserted. Random degrees of rotational malalignment were applied using a goniometer and reference wires at the fracture site. Ten physicians (consultants and residents) first estimated malrotation and then performed 150 rotational corrections: 50 according to (unstandardized) clinical practice, 50 using the lesser trochanter profile, and 50 using the true lateral view. The primary outcome was rotational malalignment, measured using reference wires at the fracture site.

**Results:**

Clinicians’ visual estimation deviated 15.5° [IQR 18.5–12.0] from actual malrotation. Corrections using unstandardized methods resulted in 12.5° [IQR 18.2–6.1] of malrotation. In contrast, the lesser trochanter and true lateral protocols reduced malrotation to 3.9° [6.8–2.1] and 3.6° [8.6–1.4], respectively. Both were significantly more accurate than current practice (*p* = 0.009 and *p* = 0.017). Surgeons favoured the lesser trochanter profile for its efficiency, ease-to-use, and minimal need for C-arm repositioning.

**Conclusion:**

This study introduces two step-by-step standardized fluoroscopy protocols to prevent rotational malalignment in femoral nailing. Both outperform traditional, unstandardized correction methods, with the lesser trochanter profile offering a more practical and time-efficient option for intraoperative use.

## Introduction

Rotational malalignment (≥ 15°) remains a common complication of intramedullary nailing of femoral shaft fractures which occurs up to 35% [1–4]. Obtaining intraoperative adequate bony alignment can be challenging, due to difficulties in interpretation of fluoroscopy images by closed reduction techniques, traction table positioning and the presence of multiple fracture fragments Besides, soft tissue injury and swelling might complicate visual interpretation of correct alignment [4–6].

Rotational malalignment of $\ge \hspace{0.17em}$15° compared to the contralateral side is considered clinically relevant and may lead to functional impairments [2, 4, 7, 8]. Moreover, it may lead to litigation according to “Guides to the Evaluation of Permanent Impairment” [9]. If remained undetected and uncorrected, patients may present with pain and loss of functioning in daily activities and sports [4, 10, 11]. Postoperatively, low-dose CT-assessment is considered the gold standard to assess malrotation, but it may reveal rotational errors that require the patient to return to theatre for revision surgery [12]. Therefore, direct intraoperative fluoroscopic protocols to identify malrotation and allow realignment would be of paramount importance. However, practical guidelines are currently lacking.

Several intraoperative fluoroscopic techniques to avoid rotational malalignment have been described, such as assessing the profile of the lesser trochanter (i.e. lesser trochanter profile) [13, 14] or the anteversion angle of the femoral neck (i.e. true lateral view) [8, 14–16]. However, these techniques have been studied on sawbones or in vitro without accounting for surrounding soft tissues and operation theatre logistics, disregarding clinical feasibility. A logistically applicable step-by-step intraoperative fluoroscopic protocol, covering patient positioning, and C-arm settings to correct rotational malalignment during intramedullary nailing following femoral shaft fractures has yet to be described.

The aim of this study is to compare the accuracy of the *lesser trochanter profile* and the *true lateral view* fluoroscopy techniques to current clinical practice of surgeons in a real operation theatre simulation using human specimens. The most accurate and clinically feasible technique will be presented as a step-by-step standardized protocol that can be used to correct rotational malalignment in intramedullary nailing of femoral shaft fractures. The protocol is designed to serve as a practical guideline for clinical practice, providing clear instructions on patient positioning, C-arm placement and orientation, and the interpretation of fluoroscopic images.

The following research questions were posed: 1. How accurate can surgeons estimate and correct femoral (mal)rotation according to current clinical practice?; 2. Can the accuracy of rotational alignment during intramedullary nailing of femoral shaft fractures be improved using the *lesser trochanter profile* or *true lateral view* fluoroscopic techniques?

## Methods

A human specimen study was conducted in the Skills Center of the University Medical Center Groningen, The Netherlands. Embalmed bodies were used. Plain radiographs were made to ensure no significant anatomical variances were present. Ten orthopaedic trauma surgeons (6 consultants and 4 residents) voluntarily participated in this study. A standard C-arm image-intensifier (GE (General Electric) OEC 9800, Salt Lake City, USA) was used to obtain fluoroscopic images. The total exposure of radiation during the experiment ranged between 0.001 and 0.003 mSv.

### Research setting

**Fig. 1:**
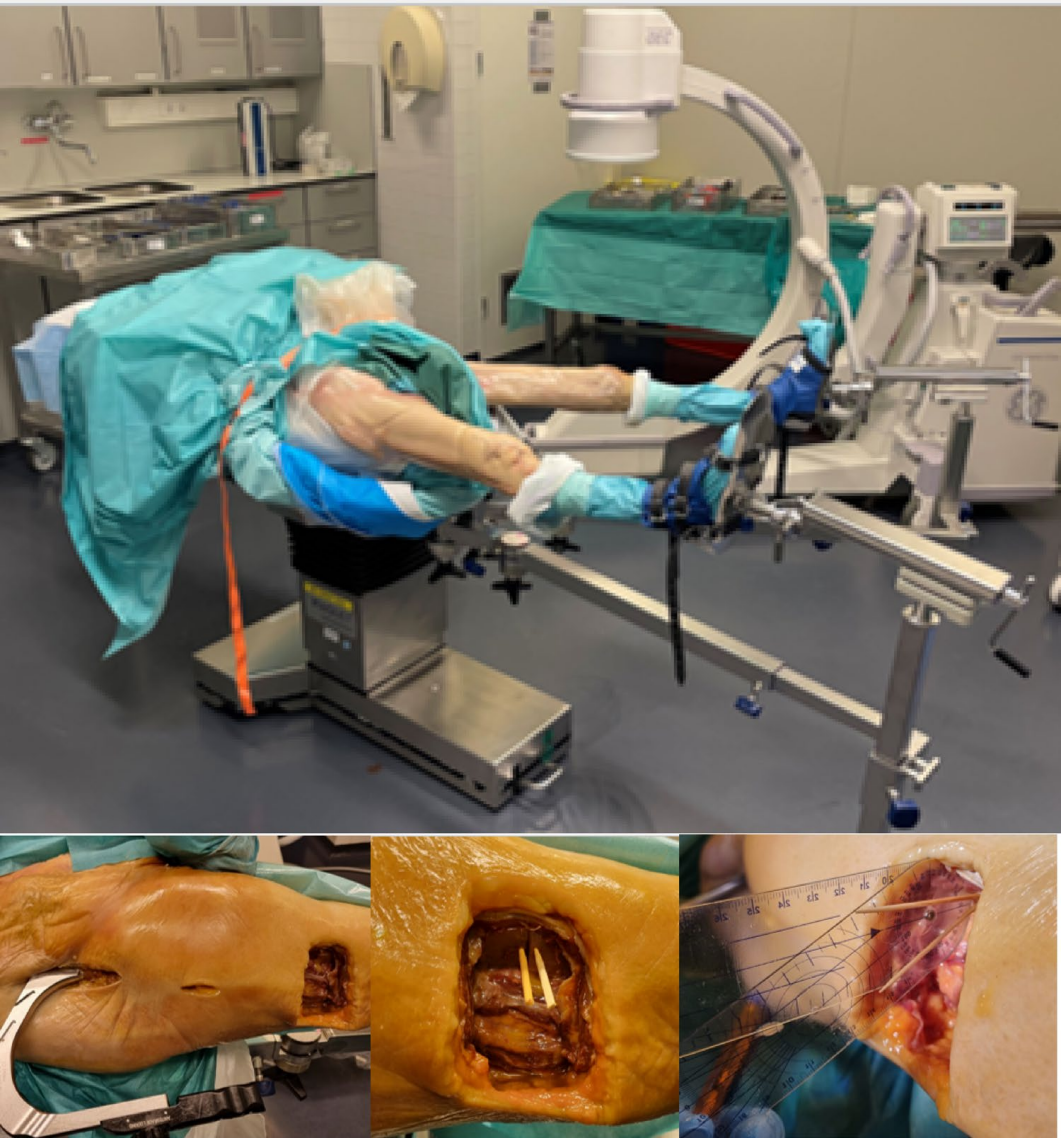
Research setting

The human specimen was positioned supine on a traction table with the right femur put under traction (Fig. 1). The left leg was put in a leg holder. Sterile draping was performed according to standard clinical practice. A transverse midshaft femoral fracture was simulated by performing an osteotomy at the midshaft of the right femur. Next, an antegrade unlocked intramedullary nail (Retrograde Antegrade Femur Nail (RAFN) 35 cm; DePuy Synthes, Switzerland) was inserted according to standard practice using a trochanteric entry. The nail was proximally locked with proximal locking screws, and the aiming device could be used to control the proximal segment. The nail was left unlocked distal to the osteotomy site. By rotating the right foot in the traction boot, various degrees of internal and external rotation of the distal femur segment could be applied. A one-mm wide hole was drilled on each side of the osteotomy site, and two parallel radiolucent (wooden) references sticks were inserted in the drilled holes to enable measuring of the alignment using a hand-held goniometer. The osteotomy and radiolucent reference wires were covered with a drape to blind the surgeons to the wooden stick positions.

### How accurate can surgeons estimate and correct femoral (mal)rotation according to current clinical practice?

Rotational malalignment between 0° and 30°, both internal and external, was randomly applied by the investigators in absence of the surgeons. The surgeons first had to estimate the applied malrotation. Then, the surgeon had to correct the applied malrotation using techniques according to their respective daily practices (e.g. based on the surgeon’s unstandardized assessment of limb positioning such as the relationship between first webspace, patella and iliac crest, and radiographic images like the cortical width), but without standardized fluoroscopic protocols. The leg was fixed in this position, the surgeon was blinded again, and the rotation was measured by one of the investigators using a hand-held goniometer to measure the angle between the radiolucent reference sticks. Each surgeon performed 5 rotational corrections without using a standardized protocol, with different degrees of external and internal rotation randomly applied by the investigators. Subsequently, five rotational corrections were performed using the *lesser trochanter profile* and lastly five using the *true lateral technique*. In total, 10 surgeons each performed 15 rotational corrections—5 without a standardized protocol, 5 using the lesser trochanter fluoroscopic protocol, and 5 using the true lateral view protocol—resulting in 150 rotational corrections overall.

These *lesser trochanter profile* and the *true lateral view* protocols were developed and are described in the subsequent paragraphs.

#### Lesser trochanter profile

**Fig. 2:**
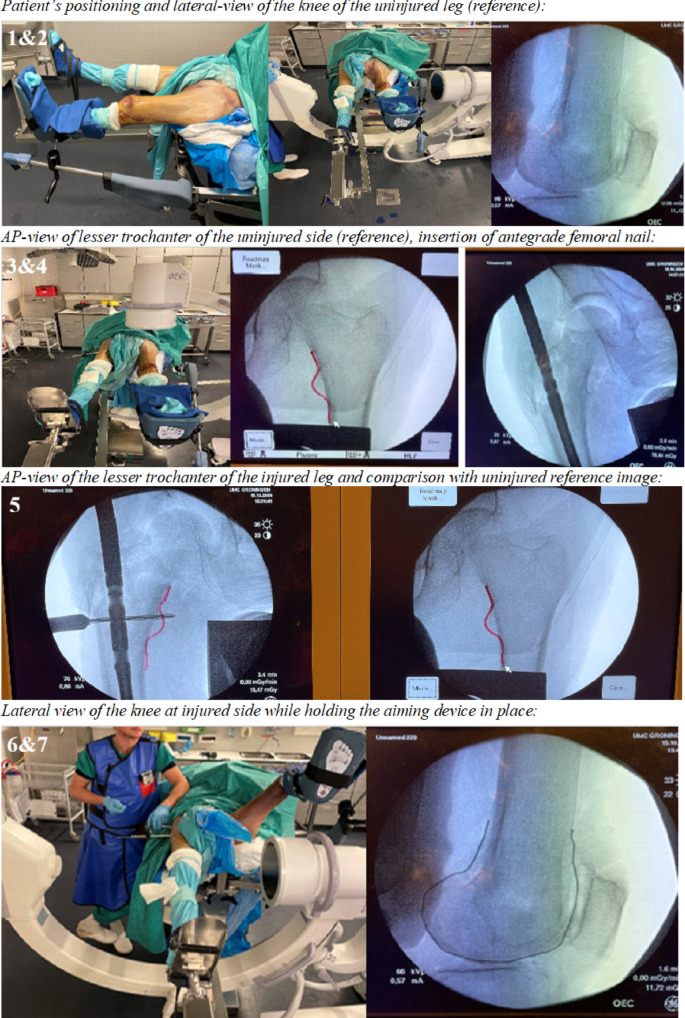
Workflow lesser trochanter profile technique. (1) Patient positioning: The patient is situated supine on a traction table. The injured leg is put in the traction device. The uninjured leg is fixed in a leg-rest. The perineal post is inserted. The injured leg is first lowered to avoid disturbance of the lateral imaging of the uninjured leg which rests straight in the leg-support. (2) Lateral-view of the knee at the uninjured leg (reference): The C-arm is positioned horizontally at the uninjured side (90° degrees on C-arm). A true lateral fluoroscopy image of the femoral condyles of the uninjured side is then obtained by rotating the leg till the condyles are superimposed while keeping the C-arm stationary. Once the optimal view is achieved, the surgeon keeps the leg in position with the assistance of the leg-rest. (3) AP-view of the lesser trochanter profile at the uninjured leg (reference) [20]: The C-arm is rotated until neutral (0°) degrees vertically and moved from the knee to the hip at the uninjured side. An anteroposterior (AP) fluoroscopy view of the uninjured lesser trochanter profile is performed and saved as a reference image. (red marking overlaid on C-arm screen to clarify procedure) (4) Insertion of femoral nail at the injured leg: An antegrade insertion of the femoral nail is performed in a standard way, and the nail is proximally locked. (5) AP-view of the lesser trochanter profile at the injured leg: With the C-arm still located on the uninjured side, an AP view of the lesser trochanter profile is obtained on the injured side. The surgeon rotates the aiming device along with the proximal femur until the fluoroscopic image precisely matches the lesser trochanter profile of the uninjured side. The investigator then held the aiming device in this exact position and secured it to the proximal fragment by temporarily placing a drill through the trocar or by inserting a proximal locking screw (6) Lateral-view of the knee at the injured leg: The C-arm is rotated to a horizontal position (90° on C-arm) and moved to the knee. A perfect lateral image of the femoral condyles is obtained by rotating the distal segment of the femur using the traction table foot, with the nail in place. Simultaneously, the proximal part is fixed in position by an assistant firmly holding the aiming device. (7) Distal locking of the femoral nail: The femoral nail is distally locked in this position. And the surgery is concluded as is usual. (8) In case ‘back-slapping’ is required to compress the fracture, proximal locking does not need to be performed first. The proximal fragment can be maintained in the correct rotational alignment using the aiming device, as the nail provides sufficient friction within the femoral shaft to prevent unintended rotation. The nail can then be locked distally, followed by back-slapping to achieve fracture compression. After back-slapping, the surgeon can recheck rotational alignment using either the lesser trochanter or true lateral technique to confirm that correct rotation has been maintained

The lesser trochanter profile technique uses the profile of the lesser trochanter in a view perpendicular to a lateral view of the femur on the healthy side as a reference. This view is subsequently replicated on the injured side to check the alignment after femoral nailing. The lesser trochanter profile technique is based on the assumption of pre-injury symmetry between both femora. This is supported by a previous study showing no substantial differences in lesser trochanter profile or femoral neck anteversion between the left and right sides [14]. A detailed workflow of the lesser trochanter technique is shown in Fig. 2.

#### True lateral technique

**Fig. 3:**
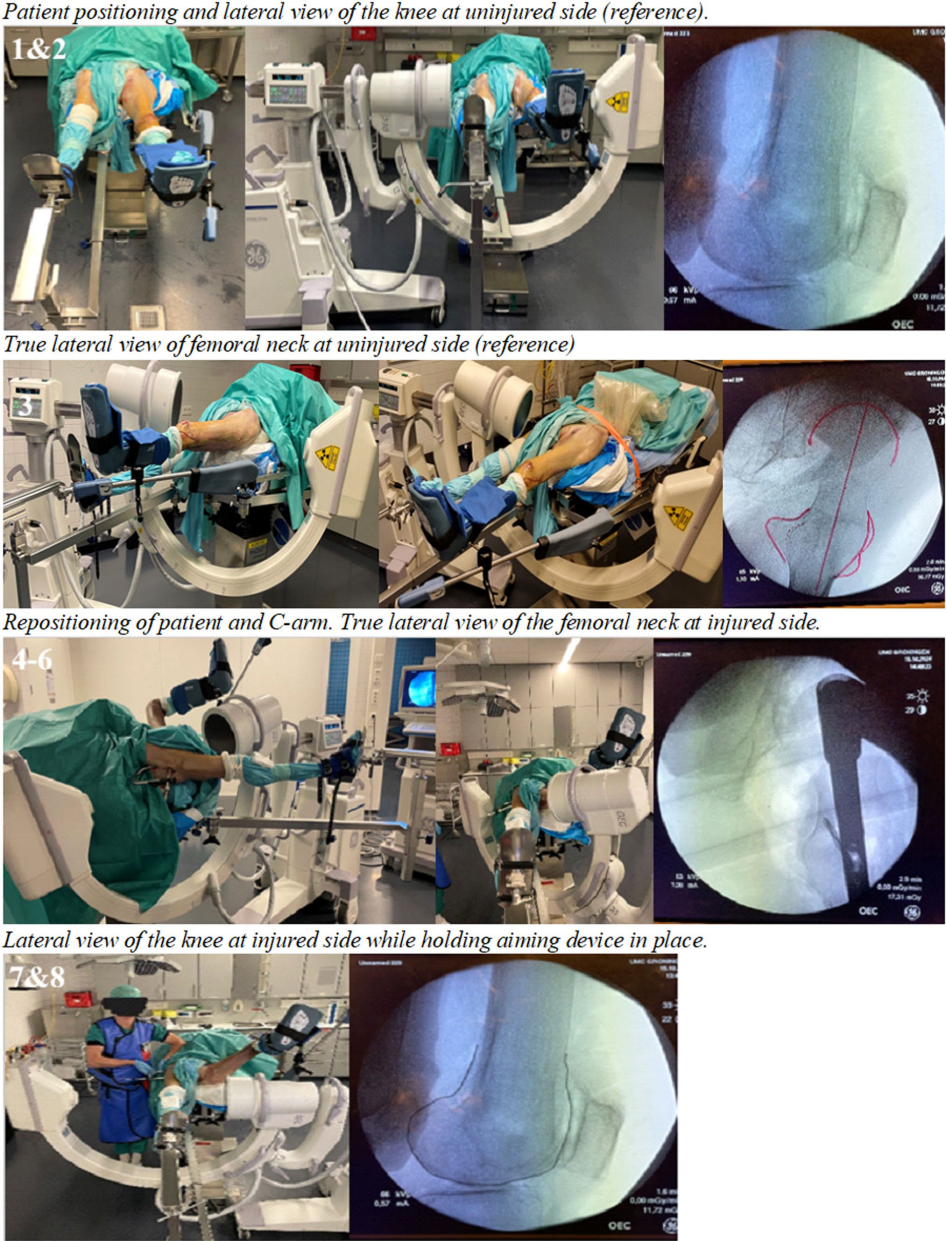
Workflow true lateral technique. (1) Patient positioning: The patient is situated supine on a traction table. The injured leg put in the traction device and the uninjured leg is fixed in a leg-rest. The perineal post is not inserted yet and the patient is situated on the table more to the uninjured side to enable fluoroscopic imaging without obscuring the image due to the table. (2) Lateral-view of the knee at the uninjured leg (reference): The C-arm is placed on the injured side in a horizontal position (90° on C-arm). The uninjured leg is rotated until a perfect lateral image of the femoral condyles is obtained. The uninjured leg is then secured in this position. (3) True lateral view of the femoral neck at the uninjured side (reference): The C-arm is moved proximally to the hip. The C-arm is carefully rotated until the femoral neck is aligned parallel to the femoral shaft, thus forming a true lateral image of the femoral neck. The angle is then verified on the C-arm and recorded. The number of degrees the C-arm has to be rotated is the anteversion angle of the femoral neck. The average anteversion angle reported in literature is 12–15° [21]. (4) Reposition of the patient: The patient is moved on the traction table to the injured side and the perineal post is situated in the usual way. The uninjured leg is situated upwards and sidewards in the leg-rest. The C-arm is moved between the patient’s legs to enable unobstructed fluoroscopy of the injured side during the procedure. (5) Insertion of femoral nail at the injured leg: An antegrade femoral nail is inserted according to the standard of care. The nail is proximally locked using the aiming device. (6) True lateral view of the femoral neck at the injured side: The C-arm is situated over the proximal femur. The anteversion angle in degrees, determined from the uninjured leg, is set on the C-arm. The proximal femur is adjusted using the aiming device until the femoral neck and shaft are parallel at the fluoroscopy images. An assistant then secured the aiming device in this position by temporarily placing a drill through the trocar or by inserting a proximal locking screw. (7) Lateral view of the knee at the injured side: The C-arm is rotated until a horizontal position (90° on C-arm) is achieved and moved to the knee. A perfect lateral image of the femoral condyles is obtained by rotating the distal part of the femur, using the traction table foot with the nail in place. Simultaneously, the proximal part is kept fixed in position by the assistant holding the aiming device. (8) Distal locking of the femoral nail: The femoral nail is distally locked in this position and the surgery is concluded as usual. (9) In case ‘back-slapping’ is required to compress the fracture, proximal locking does not need to be performed first. The proximal fragment can be maintained in the correct rotational alignment using the aiming device, as the nail provides sufficient friction within the femoral shaft to prevent unintended rotation. The nail can then be locked distally, followed by back-slapping to achieve fracture compression. After back-slapping, the surgeon can recheck rotational alignment using either the lesser trochanter or true lateral technique to confirm that correct rotation has been maintained

**Fig. 4:**
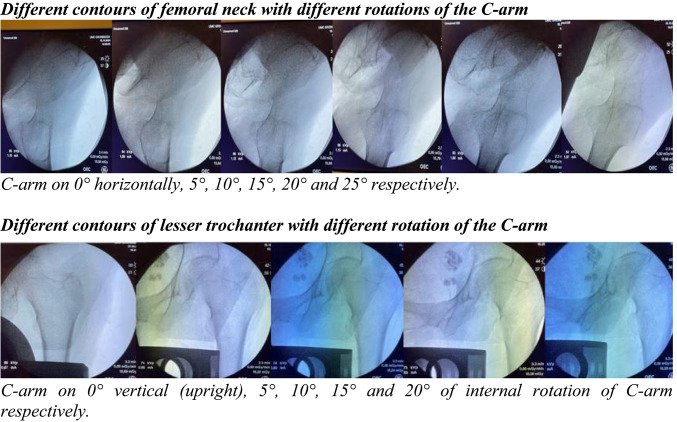
Changes in the fluoroscopic appearance of the femoral neck and lesser trochanter with varying C-arm rotation

The true lateral technique combines the anteversion angle of the femur, determined at the uninjured side, with a true lateral fluoroscopy image of the femoral condyles. A detailed workflow of the true lateral technique is shown in Figs. 3 and 4.

### How feasible are the lesser trochanter profile and true lateral technique in clinical practice?

After the measures, participants completed a questionnaire assessing the feasibility of the different techniques. The survey included questions about personal preference, impact on operation time, and overall usability in theater. An average score was attributed to each technique. A 0 being considered the worst and a 10 the best technique.

### Outcome measures

Ten orthopaedic trauma surgeons, including six consultants and four residents, performed a total of 150 rotational corrections. Fifty measures were done by using the current clinical practice at their own judgement (e.g. based on the surgeon’s unstandardized assessment of limb positioning such as the relationship between first webspace, patella and iliac crest, and radiographic images like the cortical width), 50 measures were performed using the lesser trochanter profile, and 50 measures were performed using the true lateral technique.

The primary outcome measure was the accuracy of rotational correction, assessed with a hand-held goniometer and measured in degrees at the level of the femoral fracture. Neutral rotational alignment was defined as an angle of 0° between the reference wires.

Secondary outcomes were accuracy of clinical estimation of rotation and clinical feasibility as reported by participants. The measured degrees of malrotation were categorized as either < 15°, (clinically acceptable) or $\ge$ 15°, considered not clinically acceptable malrotation.

### Statistical analysis

Statistical software package SPSS 28.0 was used for analyzing data. The rotational alignment in degrees was presented as median with interquartile ranges (IQR). Kruskal–Wallis 1-way ANOVA test was used to assess differences in alignment between the different techniques. The categorical data for acceptable and unacceptable alignment were presented as counts and percentages. Differences in malrotation correction performance between residents and consultants were evaluated for each technique using a Mann–Whitney U test. A *p* value < 0.05 was considered statistically significant.

## Results

**Fig. 5:**
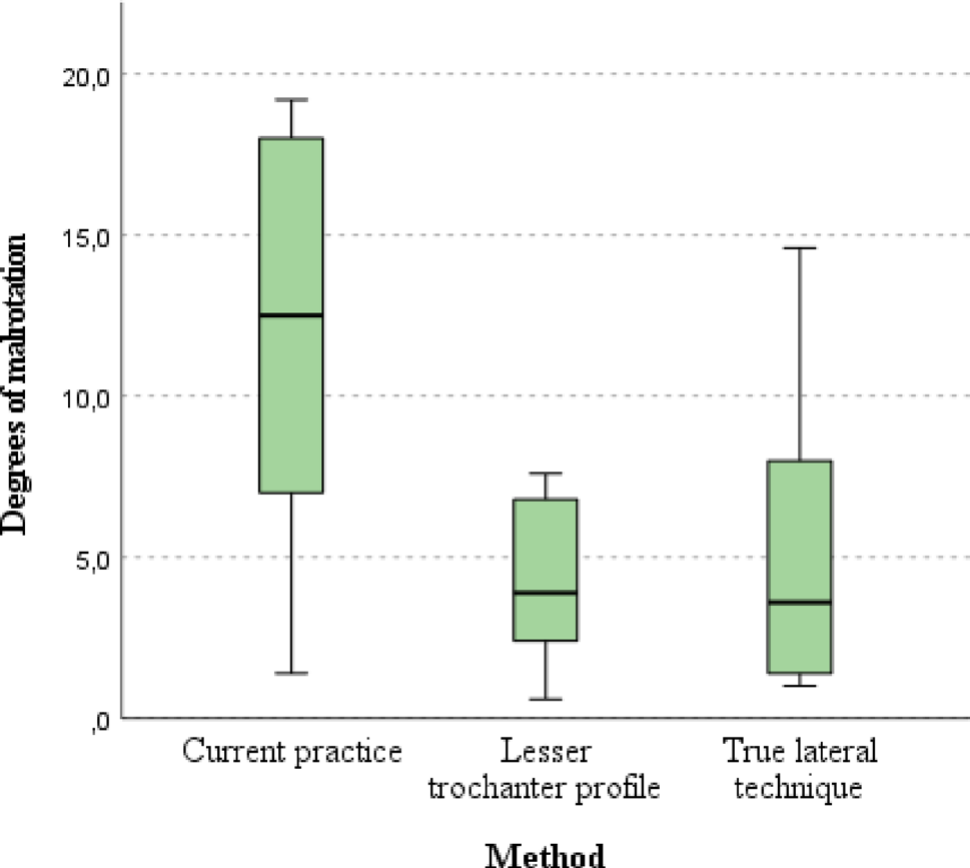
Boxplot showing median (IQR) malrotation following the use of different standardized fluoroscopy protocols to prevent malrotation in intramedullary nailing of femoral fractures

Surgeons’ clinical judgment of the (mal)rotated injured leg deviated 15.5° [IQR 18.5–12.0] from the true measured rotation that was applied. Subsequent free hand correction, using current, unstandardized clinical practice (Fig. 5), resulted in median malalignment of 12.5° [IQR 18.2–6.1]. A total of 66% resulted in acceptable rotational malalignment (< 15°) (Table 1).

**Table 1:** Overview of acceptable (< 15°) rotational malalignment using different techniques

	Current practice	Lesser trochanter profile	True lateral technique
Acceptable alignment (< 15°) (n, %)	33 (66%)	50 (100%)	48 (96%)
Unacceptable alignment (> 15°) (n, %)	17 (34%)	0 (0%)	2 (4%)

Current clinical practice of participants consisted of the following: four participants focused on fracture alignment using the C-arm, three participants focused more on the patella and the line between femoral head and first webspace of the foot. The remaining three participants used a combination of before mentioned techniques.

### Lesser trochanter profile technique

The median (mal)rotation relative to 0° after correction using the *lesser trochanter profile technique* was 3.9° [IQR 6.8–2.1] (Fig. 5). All corrected rotations were acceptable (< 15°) (Table 1).

The *lesser trochanter profile* demonstrated significantly lower median rotational malalignment compared to current clinical practice without a standardized fluoroscopic protocol (3.9° vs 12.5°; *H* = *10.25, p* = 0.009).

### True lateral technique

The median deviation from neutral alignment by using the *true lateral method* was 3.6° [IQR 8.6–1.4] (Fig. 5). A total of 96% of the corrections were acceptable (< 15°) (Table 1). The *true lateral technique* also outperformed current clinical practice with lower rotational malalignment (3.6° vs 12.5°; *H* = *9.40, p* = 0.017). The *lesser trochanter profile* group and the *true lateral technique* group performed equally well (3.9° vs 3.6° *p* = 0.83). Using either surgeons’ current clinical methods, the *lesser trochanter profile technique*, or the *true lateral technique*, 8 out of 10 participants corrected malrotation with some residual internal rotation.

### Applicability lesser trochanter profile technique & true lateral technique

On a scale from one to ten, on average the participants gave the technical applicability of the *lesser trochanter profile* an 8.2. The *true lateral technique* was deemed less technically applicable with a 6.9 on average. This was mostly the result of increased movement of the C-arm. The l*esser trochanter profile* was preferred over the *true lateral technique*, scoring an 8.1 compared to 7.1 on preference and useability in operating theatre. The participating surgeons estimated that the *lesser trochanter profile technique* required approximately 5–10 min of operating time and the *true lateral technique* about 10–15 min.

### Differences in malrotation correction performance between consultants and residents

Significant differences in rotational alignment were found between residents and consultants using current clinical practice (*p* = 0.03). However, using respectively the *lesser trochanter profile* and the *true lateral technique*, no significant difference in residual rotation was found (*p* = 0.09 and *p* = 0.52).

## Discussion

Rotational malalignment following intramedullary fixation of femoral fractures is common. Our study confirmed the difficulty in obtaining correct rotational alignment as well as the inaccuracy of current unstandardized clinical standards. We therefore introduced a step-by-step standardized approach towards increased accuracy of intra-operative rotational control using the *lesser trochanter profile* or *true lateral* technique.

Our first research question focused on the estimation and correction of rotational malalignment according to current clinical standards, encompassing methods from visual inspection of leg alignment to fluoroscopic evaluation of cortical width at the fracture site. The estimated malrotation differed on average 15.5° from the applied malrotation and correction of malrotation resulted in a median malalignment of 12.5°. Our study adds to current literature that clinical assessment and unstandardized correction of rotational alignment during nailing of femoral fractures is often inadequate.

Our second question focused on the reduction of rotational malalignment using the *lesser trochanter profile* and the *true lateral technique* as standardized intraoperative fluoroscopy protocols. Both the *lesser trochanter profile* and the *true lateral* technique improved the accuracy of rotational alignment during intramedullary fixation of femoral fractures. In 1995, Tornetta et al. [18] reported on the challenge of accurately restoring femoral rotation during intramedullary nailing, especially in cases with comminuted fractures. They introduced an intraoperative fluoroscopic technique that reproduces the normal femoral anteversion. The technique involves first determining the normal femoral anteversion by obtaining a true lateral view of the uninjured hip and aligning the posterior condyles at the knee using the image intensifier to measure the rotational angle. This angle is then replicated on the fractured side by obtaining a true lateral of the injured hip and rotating the C-arm at the distal femur until the posterior condyles align with the previously measured angle. Our technique differs slightly from that of Tornetta et al. We start with a true lateral of the knee, then obtain a true lateral of the hip, recording the C-arm rotation angle to objectively determine the anteversion. In contrast, Tornetta et al. begins with a true lateral of the hip and estimates anteversion based on the alignment of the femoral condyles. The advantage of our true lateral technique is that we objectively measure the anteversion angle using the rotation degree on the C-arm, whereas Tornetta et al. rely on estimation based on fluoroscopic images.

In 1998, Deshmuck et al. [19] were one of the first that described the technique relying on the lesser trochanter profile in a small cohort study of ten patients. They found that adhering to this standardized protocol significantly reduced rotational malalignment compared to the unstandardized clinical practice. However, their lesser trochanter profile technique differs slightly from ours, as they determine the position of the distal femur clinically. This approach may lead to inaccuracies, whereas in our study, distal femoral alignment was objectively assessed using a true lateral image of the knee. Kim et al. [20] underline these findings. Recently, Ivanov et al. [8] performed a cadaveric study comparing fluoroscopy-based techniques for estimating femoral rotation. They evaluated both the lesser trochanter profile and true lateral techniques for correcting rotational alignment of the injured femur, using the contralateral intact femur as a reference. Their results showed promising accuracy, with absolute mean rotational errors of 8.5° for the lesser trochanter profile and 6.0° for the true lateral technique. However, direct comparison with our findings is challenging, as their study involved fully dissected femora with the surrounding soft tissue envelope removed. Additionally, they did not use implants or replicate a clinical setting. In contrast, our study simulated a realistic clinical environment, including soft tissues and implants, thereby demonstrating the clinical feasibility of fluoroscopy-guided techniques in a step-by-step manner that can be applied by clinicians worldwide.

In our study, we performed a short survey among participants about applicability of fluoroscopy protocols in theatre. The *lesser trochanter profile* method was generally preferred because it requires less time to perform and allowed both the C-arm and patient to remain in position. In contrast, the true *lateral technique* was estimated to add an additional 15 min to the surgical procedure due to the need for patient repositioning and C-arm adjustment. From a clinical perspective, participants regarded the true lateral technique less practical than the lesser trochanter profile technique.

This study has some strengths and some limitations. One of the strengths is the realistic simulation of an operation theatre environment rather than testing fluoroscopy protocols on isolated (saw)bones, allowing assessment of intraoperative feasibility for both the surgeon as the radiology technician. Additionally, since participants were surgeons regularly performing intramedullary fixation of femoral fractures, the findings and answers on the feasibility questionnaire can be considered representative. Furthermore, no significant differences in malrotation correction performance were observed between residents and consultants when using either the lesser trochanter profile or the true lateral technique. In contrast, using current clinical practice, there was a significant difference in residual rotation between residents and consultants. These findings suggest that the implementation of a standardized fluoroscopic protocol may partially compensate for differences in surgical experience. Consequently, even less-experienced residents may be able to achieve clinically acceptable rotational correction when guided by a strict imaging protocol. The following limitations should be considered. First, the fluoroscopy protocols were only tested in a human specimen with a transverse midshaft fracture, other fracture patterns were not evaluated. While this may limit generalizability to other fracture types, transverse fractures are in fact the fracture pattern prone to malrotation. However, since both techniques rely on reference fluoroscopic images obtained proximal and distal to the fracture site, we believe this limitation does not disqualify our findings. Second, the procedure was performed using an antegrade nail, which may not fully reflect the variety of clinical scenarios. Although not tested, both protocols could theoretically be applied to retrograde nailing as well. Finally, no formal sample size calculation was performed. As no previous studies have evaluated these fluoroscopic techniques in a clinical or experimental setting, it was not possible to reliably estimate an expected effect size or variability from the literature. We therefore adopted a pragmatic study design in which 10 surgeons each performed 15 rotational corrections—5 without a standardized protocol, 5 using the lesser trochanter protocol, and 5 using the true lateral protocol—resulting in a total of 150 measurements. This number was considered practically feasible and sufficient to allow a meaningful comparison between the techniques.

## Conclusion

This study showed that strict adherence to a standardized intraoperative fluoroscopy protocol significantly reduces the rotational malalignment during intramedullary nailing of femoral fractures. We provide a clear, step-by-step guide for using the lesser trochanter profile and true lateral view to improve rotational alignment compared to non-standardized methods, which are often inaccurate. Surgeons preferred the lesser trochanter profile technique because it is more practical. Although the fluoroscopy protocols can be directly applied in theatre, a clinical trial to confirm their effectiveness in reducing rotational malalignment in patients with femoral fractures is yet to be conducted.

## Data Availability

The data is available on reasonable request from the corresponding author.
